# Short-Term High-Fat Feeding Does Not Alter Mitochondrial Lipid Respiratory Capacity but Triggers Mitophagy Response in Skeletal Muscle of Mice

**DOI:** 10.3389/fendo.2021.651211

**Published:** 2021-03-31

**Authors:** Sarah E. Ehrlicher, Harrison D. Stierwalt, Sean A. Newsom, Matthew M. Robinson

**Affiliations:** School of Biological and Population Health Sciences, Oregon State University, Corvallis, OR, United States

**Keywords:** high-fat feeding, reactive oxygen species, respiration, autophagy, mitochondria

## Abstract

Lipid overload of the mitochondria is linked to the development of insulin resistance in skeletal muscle which may be a contributing factor to the progression of type 2 diabetes during obesity. The targeted degradation of mitochondria through autophagy, termed mitophagy, contributes to the mitochondrial adaptive response to changes in dietary fat. Our previous work demonstrates long-term (2-4 months) consumption of a high-fat diet increases mitochondrial lipid oxidation capacity but does not alter markers of mitophagy in mice. The purpose of this study was to investigate initial stages of mitochondrial respiratory adaptations to high-fat diet and the activation of mitophagy. C57BL/6J mice consumed either a low-fat diet (LFD, 10% fat) or high-fat diet (HFD, 60% fat) for 3 or 7 days. We measured skeletal muscle mitochondrial respiration and protein markers of mitophagy in a mitochondrial-enriched fraction of skeletal muscle. After 3 days of HFD, mice had lower lipid-supported oxidative phosphorylation alongside greater electron leak compared with the LFD group. After 7 days, there were no differences in mitochondrial respiration between diet groups. HFD mice had greater autophagosome formation potential (Beclin-1) and greater activation of mitochondrial autophagy receptors (Bnip3, p62) in isolated mitochondria, but no difference in downstream autophagosome (LC3II) or lysosome (Lamp1) abundance after both 3 and 7 days compared with the LFD groups. In cultured myotubes, palmitate treatment decreased mitochondrial membrane potential and hydrogen peroxide treatment increased accumulation of upstream mitophagy markers. We conclude that several days of high-fat feeding stimulated upstream activation of skeletal muscle mitophagy, potentially through lipid-induced oxidative stress, without downstream changes in respiration.

## Introduction

Skeletal muscle contains a high abundance of mitochondria that govern oxidative metabolism and are implicated in the progression of type 2 diabetes during obesity (1). Diet-induced obesity from high-fat over-feeding increases lipid substrate availability for mitochondrial oxidative metabolism. The lipid overload of the mitochondria is linked to the development of insulin resistance in mice (2) and humans (3, 4). However, it is not understood how lipid overload drives alterations in mitochondrial function. Such knowledge is necessary for understanding points of dysregulation during progression from insulin resistance to type 2 diabetes.

High-fat feeding in mice is a common model of insulin resistance that also induces mitochondrial adaptations. High-fat over-feeding in mice induces whole-body glucose intolerance within 3 days and skeletal muscle insulin resistance by 3 weeks (5), demonstrating a rapid response in skeletal muscle to high-fat feeding. Long-term (3-4 months) diet studies with increased dietary lipid availability increases mitochondrial fatty acid oxidation capacity (6) due to increases in mitochondrial protein abundance (7, 8) and enzyme activity (9). However, alterations in the mitochondrial regulatory pathways within a shorter time frame (< 1 week) are not as well characterized. Characterizing the early time-course (< 1 week) of changes in mitochondrial regulatory pathways is necessary for understanding the early events that contribute to mitochondrial adaptations to lipids that can result in increased respiratory capacity.

A primary pathway of mitochondrial quality control regulation is targeted degradation of proteins through autophagy, termed mitophagy. Autophagy is chronically active to maintain the quality of mitochondria in skeletal muscle (10) and is upregulated in response to stress such as exercise or injury (11, 12). Disruption of autophagy in mice leads to abnormal skeletal muscle mitochondria with increased oxidative damage and impaired respiratory function (13). Two predominant mitophagy mechanisms include Parkin, an E3 ligase, that ubiquitinates proteins on the mitochondrial membrane for recruiting autophagosomes (14) and Bcl-2/adenovirus E1B interacting protein 3 (Bnip3), an autophagosome receptor that resides on the outer mitochondrial membrane (15).

In skeletal muscle, fatty acid oxidation generates reactive oxygen species (ROS) in the mitochondria (16, 17), which play a role in biological signaling (18) and stimulate mitophagy (19, 20). Previous work, focused on heart tissue, demonstrated that high-fat diet induces mitophagy in a Parkin-dependent manner, as a protective mechanism against diabetic cardiomyopathy (21). Our previous work in mice demonstrated Bnip3, but not Parkin-mediated mitophagy, was upregulated in skeletal muscle after long-term high-fat feeding (6), suggesting mitophagy is a contributing mechanism to mitochondrial adaptations to lipid overload. Lipid-induced mitochondrial ROS production may be a contributing stimulus of mitophagy mechanisms during initial mitochondrial adaptations to high-fat feeding.

The purpose of this study was to investigate initial stages of mitochondrial respiratory adaptations to high-fat diet and the activation of mitophagy. We hypothesized that short-term high-fat feeding would increase lipid oxidation and stimulate mitophagy. To address this hypothesis, mice were fed a 60% high-fat diet (HFD) for 3 or 7 days to measure the mitochondrial adaptive response. The rationale for short-term high-fat feeding was to challenge the mitochondria with lipid overload and measure the mitochondrial stress response prior to longer-term adaptations over weeks or months. We used C2C12 myotubes to further examine the impact of lipid overload or oxidative stress on mitochondrial regulation by measuring autophagy flux and mitochondrial membrane potential. Our primary findings indicate that, compared to low-fat diet (LFD) fed mice, mice fed HFD for 3 days had greater mitochondrial ROS production with no changes to maximal respiratory capacity. HFD groups had greater Bnip3-mediated mitophagy compared to LFD. Myotube studies demonstrated greater Bnip3 abundance after lipid treatment compared to control, supporting mitophagy as a stress response to high lipids. We conclude that short-term consumption of HFD increases ROS production and greater Bnip3-mediated mitophagy as part of an adaptive stress response to lipid overload.

## Methods

### Animals and Diets

Male C57BL/6J mice were purchased from Jackson Laboratories (Bar Harbor, ME, USA) at eight to ten weeks of age. The genotype and age range has rapid weight gain on a high-fat diet. Mice were housed five per cage in 12:12-h light-dark cycle at 22°C with free access to food and water and allowed to acclimate for at least one week before beginning respective diets. The animal study protocol was approved by the Animal Care and Use Committee at Oregon State University (ACUP #4951). Group numbers were n=5 per diet and time point.

Mice consumed either a low-fat diet (LFD; D12450J; Research Diets) or high-fat diet (HFD; D12492; Research Diets) ad libitum for 3 days and 7 days to measure acute effects on mitochondrial respiration. A third group consumed diets for 12 weeks to investigate long-term changes in protein abundance and signaling, but not respiration because long-term changes in respiration have been well-documented by our group (6, 22). The percent kilocalories from total fat (from lard)/carbohydrate/protein was 10/70/20 for the LFD and 60/20/20 for the HFD. The diets were matched for sucrose content (7% of kilocalories) and contain similar micronutrients and vitamins (but vary in cholesterol). The HFD was the intervention of interest and we used LFD as a control to account for stress induced in the mice from changes in diet from chow.

After the last day of the diet intervention, mice were fasted for four hours then body weight was recorded and blood glucose concentration was measured by handheld glucometer (Alphatrak2, Zoetis) from a tail vein. Mice were anesthetized with sodium pentobarbital overdose through an intraperitoneal injection and euthanized with cardiac puncture then cardiac excision. Whole blood from cardiac punctures were transferred to EDTA tubes then centrifuged to collect plasma for measuring insulin concentrations (Insulin ELISA, Alpco). Tissues were extracted and snap-frozen in liquid nitrogen.

### Mitochondrial Preparation and Respiration

Mitochondria were isolated using differential centrifugation of freshly dissected quadriceps muscle as described (23). Quadricep muscle (~100 mg) was incubated in buffer A (100 mM KCl, 50 mM Tris base, 5 mM MgCl_2_-6H_2_O, 1.8 mM ATP, and 1 mM EDTA, pH 7.2) containing protease (Subtilisin A, P5380, Sigma-Aldrich, St. Louis, MO, USA) for 7 minutes on ice then homogenized in glass-on-glass homogenizers with 0.3-mm spacing between mortar and pestle for 10 minutes at 150 rpm. Samples were centrifuged for 5 minutes at 750 × *g* and 4°C to collect the myofibrillar proteins. The supernatant was centrifuged for 5 minutes at 10,000 × *g* and 4°C to pellet the mitochondria. The supernatant was discarded and the mitochondrial pellet was washed with buffer A and centrifuged 5 minutes at 9,000 × *g* and 4°C. The supernatant was discarded and the mitochondria were resuspended in 1:4.2 (wt/vol) buffer B (180 mM sucrose, 35 mM KH_2_PO_4_, 10 mM Mg-Acetate, 5 mM EDTA, pH=7.5).

High-resolution respirometry was performed using Oxygraph-2k units (Oroboros Instruments, Innsbruck, Austria) with MiR05 respiration buffer (0.5 mM EGTA, 3 mM MgCl_2_-6H_2_O, 60 mM lactobionic acid, 20 mM taurine, 10 mM KH_2_PO_4_, 20 mM HEPES, 110 mM sucrose, and 1 g/L bovine serum albumin). We measured oxygen consumption and hydrogen peroxide (H_2_O_2_) emission (10 μM Amplex red, 5 U/ml superoxide dismutase and 1 U/ml horseradish peroxidase) in duplicate chambers (A and B). The rates of oxygen consumption (JO_2_; pmol O_2_/sec/mL) and H_2_O_2_ emission (JH_2_O_2_; pmolH_2_O_2_/sec/mL) at each point were calculated as the average values from the A and B chambers. Protein concentration of the mitochondrial preparation was measured by Pierce BCA assay (Thermo Fisher Scientific, Waltham, MA, USA) to calculate JO_2_ relative to protein content (pmol O_2_/μg protein/sec). Since the rates of O_2_ consumption and H_2_O_2_ emission were measured simultaneously, we were able to calculate electron leak to ROS as a fraction of total O_2_ consumption (JH_2_O_2_/(2xJO_2_)) (24).

The protocol consisted of sequential additions of substrates and inhibitors to assess oxidative phosphorylation, leak and uncoupled state respiration. Addition of the mitochondrial suspension (90 μl) was followed by ADP (5 mM), malate (0.1 mM), palmitoyl-carnitine (0.025 mM), malate (2 mM), octanoyl-carnitine (0.5 mM), pyruvate (5 mM), glutamate (10 mM), succinate (10 mM) and glycerophosphate (1 mM) to measure oxidative phosphorylation state respiration. Cytochrome c (10 μM) was added to test the membrane integrity of the mitochondrial prep. The complex V inhibitor, oligomycin (2.5 μM), was added next to inhibit ATP synthesis and measure leak state respiration. The chemical uncoupler, carbonyl cyanide p-(trifluoromethoxy) phenlyhydrazone (FCCP) was titrated with 0.5 μM additions until maximal uncoupled state respiration was reached. The complex I inhibitor, rotenone (0.5 μM), was added to measure the contribution of complex II to uncoupled respiration. Lastly, antimycin A (2.5 μM) was added to inhibit complex III and measure non-mitochondrial oxygen consumption.

### Immunoblotting

Quadriceps and gastrocnemius muscles were processed as whole tissue homogenates (~40 mg) or mitochondrial isolations (~70 mg) for immunoblotting as previously described (22). Approximately 35 μg of protein was resolved on 10-15% Bis-Tris gels and transferred to nitrocellulose membranes. Membranes were blocked in 5% bovine serum albumin (BSA) in Tris-buffered saline + 1%Tween (TBST). Primary antibodies were diluted in 5% BSA-TBST or 5% non-fat dry milk-TBST and membranes incubated overnight at 4°C.

Primary antibodies for OXPHOS cocktail (cat#ab110413), Mfn2 (cat#ab56889), Mff (cat#ab81127) and Vdac (cat#ab14734) were purchased from Abcam (Cambridge, UK). Primary antibodies for LC3 (cat#12741), p62 (cat#39749), Parkin (cat#4211), Bnip3 (cat#3769), Ulk1 pSer555 (cat#5869), Beclin-1 (cat#3495) and Drp1 (cat#14647), were purchased from Cell Signaling Technology (Danvers, MA, USA). Primary antibody for Hadh (cat#PA5-28203) was purchased from Thermo Fisher Scientific (Waltham, MA, USA). Secondary antibodies were purchased from Licor (Lincoln, NE, USA), diluted in 5% BSA-TBST or 5% non-fat dry milk-TBST and membranes incubated at room temperature for 1-hour.

Ponceau staining of membranes was performed to verify equal loading and transfer of protein to the nitrocellulose membrane. After Ponceau staining, membranes were cut at pre-determined molecular weights to probe for multiple targets from a single membrane without the need for stripping between multiple antibodies. Images were generated using infrared detection (Odyssey, Licor) and analyzed using ImageView software (Licor). A control sample was loaded at the beginning and end of each gel to serve as an internal control. The average intensity of the internal control for each protein target was used to normalize band intensities to account for any methodological variation between gels. We used Vdac as a marker of mitochondrial enrichment in the mitochondrial fractions. A representative blot image of Vdac is presented with each protein target generated from the same membrane.

### Cell Culture Methods

*In vitro* experiments were used to provide a reductionist approach for measuring the effects of lipid or oxidative stress on autophagy activation and determine mechanisms that were not feasible during the *in vivo* study. C2C12 myoblasts (CRL-1772; ATCC, Manassas, Virginia, USA) were grown in Dulbecco’s modified Eagle’s medium (DMEM, Gibco) supplemented with 1% antibiotic/antimycotic (Gibco) and 10% fetal bovine serum (FBS, ATCC) in a 5% CO_2_ humidified atmosphere at 37°C and seeded to 10 cm plates. Once cells reached ~80-90% confluence, differentiation into myotubes was induced by switching to low glucose DMEM supplemented with 1% antibiotic/antimycotic and 2% horse serum for 4-5 days.

#### Fatty Acid Treatments

Fatty acid treatment was used to provide a direct lipid stress on myotubes. We used two different lipid treatments to test if the mitochondrial response was specific to representative lipid species (palmitate or palmitate plus oleate). Fatty acid treatments were prepared in 100% ethanol and then diluted in culture media supplemented with 2% bovine serum albumin and 1 mM carnitine with a final concentration of 0.5% ethanol. Myotubes were treated with 300 μM palmitate (palmitic acid; Sigma-Aldrich) or the combination of palmitate and oleate (oleic acid; Sigma-Aldrich) at a ratio of 1:3. Control cells were treated with 0.5% ethanol in BSA and carnitine supplemented media as a vehicle control. Autophagosome degradation was inhibited with 100 nM bafilomycin (Sigma-Aldrich) treatment for the duration of the fatty acid stimulus. Fatty acid treatments were performed for 4, 8 or 24 hours to determine if changes in autophagy were transient. Three experiments each with two replicates were performed from separate cell stocks to generate a total sample size of n=6 for each treatment and timepoint.

#### Hydrogen Peroxide Treatments

Hydrogen peroxide (H_2_O_2_) treatment was used to provide a direct oxidative stress on myotubes. H_2_O_2_ (Sigma-Aldrich) was diluted in media to final concentrations (0.5 mM or 5 mM) immediately before treatment. H_2_O_2_ concentrations were chosen based on a previous report of decreased mitochondrial membrane potential and increased localization of LC3 to the mitochondria in C2C12 myotubes (25). Bafilomycin was added 30 minutes before H_2_O_2_ was present and for the duration of the 1-hour H_2_O_2_ treatment. Three experiments each with two replicates were performed from separate cell stocks to generate a total sample size of n=6 for each treatment.

#### Mitochondrial Fractionation for Immunoblotting

Myotubes were harvested in 250 μl of mitochondrial isolation buffer A plus protease inhibitor cocktail. The cell lysates were homogenized with a handheld pulse homogenizer for 20 seconds then centrifuged for 15 minutes at 10,000 × *g* to pellet mitochondria. An initial low speed spin was not conducted in the muscle cells as in the muscle tissue because of the lower sample available for yielding sufficient amounts of mitochondrial protein for immunoblotting methods. The supernatant was saved in a separate tube and the mitochondrial enriched pellet was resuspended in 200 μl lysis buffer + 100 μl 8 M urea. Protein concentrations of the supernatant and pellet were determined by bicinchoninic acid assay (BCA, Thermo Fisher) and samples were analyzed by immunoblotting as described above.

#### Mitochondrial Membrane Potential

Myoblasts were seeded to a 96-well plate (~3000 cells/well) and differentiated once cells reached ~90% confluence. Treatment conditions are the same as described above and were run in triplicate where the presented data are an average of the three wells relative to the average of the control. Multiple experiments on separate plates and from separate cell stocks were performed to generate a total sample size of n=4-11. The negative control was DMED and the positive control was 10 μM FCCP treatment for 24 hours. At the end of the time point, treatment media was removed and DMED with 5 μM JC-1 probe was added (Molecular Probes/ThermoFisher, cat# T3168) for 30 minutes. At the end of the incubation, JC-1 probe was removed, the wells were washed twice with warmed sterile PBS and fresh DMED was added to the wells. Fluorescence was measured on a spectrophotometer at 535 excitation/590 emission (red) and 485 excitation/530 emission (green) and the data are expressed as the ratio of red/green signals.

### Statistical Analysis

Planned comparisons between groups were performed using unpaired t-tests or two-way analysis of variance model (ANOVA) by diet and time, as specified in figure legends. Repeated measures ANOVA and Tukey’s post-hoc tests were performed to detect differences between respiration states in the mitochondrial respiration data and corrected for multiple comparisons. Statistical significance was set as *p* < 0.05. Statistical analysis was performed using Prism version 6 (GraphPad Software, La Jolla, CA, USA). Data are expressed as means with SD and group sizes are reported in figure legends. Main effects from the ANOVA tests are presented in the figures with p values when the results reached or were close to reaching statistical significance.

Based on our previous report of HFD and LFD feeding in the same strain of mice, lipid respiration normalized for mitochondrial protein abundance for LFD mice was Mean (SD) of 1.3 (0.38) and HFD was 1.8 (0.76) (6). Using n=5 per group, SD of 0.4, α at 0.05 and 1-β of either 0.8 or 0.5, an un-paired t-test could detect differences of 0.81 and 0.56. We were powered to detect effects sizes of 2 and 1.4, which aligns with effect sizes of HFD from our previous work that demonstrated physiologically significant outcomes.

## Results

### Phenotypic Characteristics

The purpose of the short-term high fat diet was to challenge the mitochondria with lipid overload. The high-fat diet fed (HFD) mice gained more weight compared with the low-fat diet fed (LFD) mice after both 3 and 7 days (**Table 1**). Fasting blood glucose of the HFD mice was greater than the LFD mice after 3 days (+43%, p<0.01, t-test) but not 7 days (+3%, p=0.87, t-test) (**Table 1**). Fasting plasma insulin was not different between LFD or HFD mice. Overall, fasting hyperglycemia and increases in body weight were evident at 3 days of high-fat diet without elevations in plasma insulin. The phenotype suggests the lipid challenge caused slight disruptions to whole-body glucose metabolism. We next tested the effect of the acute lipid challenge on mitochondrial respiratory function.

**Table 1:** Mice characteristics after short-term high-fat diet. P values are from un-paired t-tests for low-fat diet (LFD) compared to high-fat diet (HFD). Data are mean (SD) with n = 5 per diet group.

	3-Day			7-Day		
	LFD	HFD	P Value	LFD	HFD	P Value
Body weight change (g)	-1.7 (0.5)	1.2 (0.3)	<0.001	-1.5 (0.6)	2.0 (1.4)	<0.001
Blood glucose (mg/dl)	215 (16)	307 (30)	<0.01	198 (17)	204 (79)	0.87
Plasma insulin (ng/ml)	0.56 (0.17)	0.58 (0.19)	0.92	0.69 (0.23)	0.51 (0.15)	0.33

### Short-Term High-Fat Feeding Alters Mitochondrial Lipid Respiration

**Figure 1:**
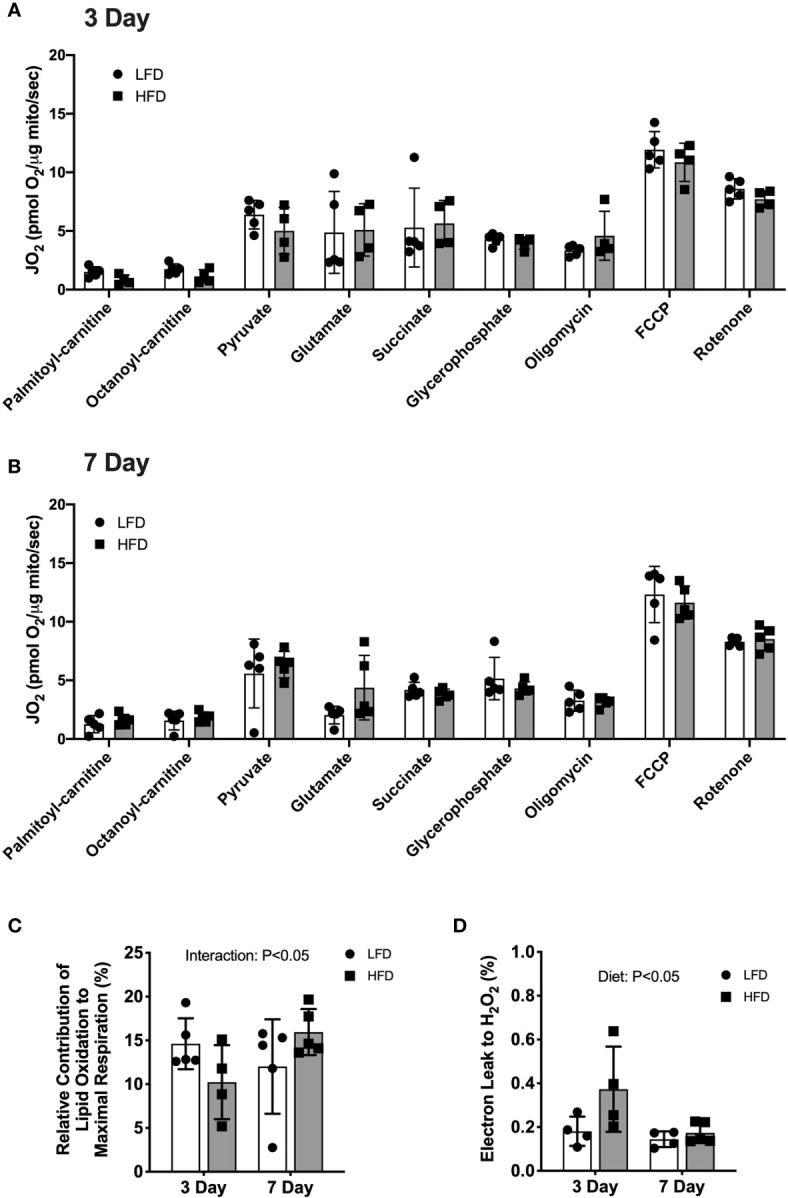
3 days of high-fat feeding lowered lipid substrate contribution to respiration and stimulated reactive oxygen species production. Mitochondrial respiration of isolated mitochondria from quadriceps muscle during sequential additions of substrates and inhibitors. **(A)** Rates of oxygen consumption expressed relative to protein content (pmol O_2_/μg protein/sec) from mice fed low-fat or high-fat diet for 3 days. **(B)** Rates of oxygen consumption expressed relative to protein content (pmol O_2_/μg protein/sec) from mice fed low-fat or high-fat diet for 7 days. **(C)** Contribution of lipid-supported oxidation expressed as a percentage of maximal respiration measured after FCCP. **(D)** Electron leak to H_2_O_2_ calculated from H_2_O_2_ emission during lipid-supported oxidative phosphorylation and expressed as a percentage of oxygen consumption. Data are means ± SD; n=4-5. Repeated measures ANOVA with Tukey’s post-hoc tests were used to analyze data in **(A, B)**. Two-way ANOVA was used to test for effects of diet and time in **(C, D)**. P values are main effects.

We used high-resolution respirometry on isolated mitochondria to test the capacity for substrate oxidation after short-term high-fat feeding. The protocol consisted of sequential additions to provide electrons from lipid and non-lipid substrate oxidation. No significant differences in respiration rates between the diet groups were detected at either 3 days or 7 days (**Figures 1A, B**). In the 3-day group, the respiration rates increased with the addition of pyruvate compared with palmityl-carnitine (**Figure 1A**). However, the addition of glutamate, succinate or glycerophosphate did not increase respiration rates above pyruvate (**Figure 1A**). The addition of the ATP synthase inhibitor, oligomycin, induced leak respiration that was similar to that during oxidative phosphorylation (**Figure 1A**). The 7-day mice had a similar pattern of respiration rates increasing after the addition of palmityl-carnitine and pyruvate but did not increase further with glutamate, succinate or glycerophosphate (**Figure 1B**). Multiple substrates maximize entry of electrons into the electron transfer system, but the relative contribution of each fuel source cannot be separated. The lack of stimulation may indicate a lower respiration of combined fuel sources relative to individual substrates which is consistent with substrate overload reported previously (2). Respiration increased after the addition of the chemical uncoupler, FCCP (**Figures 1A, B**), indicating no restriction in electron transfer system capacity. These data demonstrate that short-term feeding of a high-fat diet does not alter maximal lipid or non-lipid supported respiratory capacity. We have reported significant changes in lipid-supported respiration after 12 weeks of HFD (6, 22) suggesting longer durations are required for diet-induced increases in respiratory capacity.

There was a diet × time interaction (p<0.05) such that lipid-supported respiration (PC + OC) was a lower percentage of maximal uncoupled respiration (FCCP) after 3 days of HFD (-30%) but a higher percentage after 7 days of HFD (+32%) compared with LFD (**Figure 1C**). The relative contribution of non-lipid supported respiration to maximal respiration was not different between diet groups (data not shown), suggesting the alterations in oxidative phosphorylation were specific to lipid substrates. The electron leak to hydrogen peroxide production during lipid-supported respiration was higher after 3 days of HFD (+106%, p<0.05) compared with LFD (**Figure 1D**). No significant difference between diet groups was detected after 7 days of diet intervention (**Figure 1D**). These data demonstrate that 3 days of high-fat diet is a stress on the mitochondria with lower lipid-supported oxidative phosphorylation alongside greater electron leak to hydrogen peroxide. Such differences were not observed after 7 days of high-fat diet, suggesting a transient alteration in mitochondrial function.

**Figure 2:**
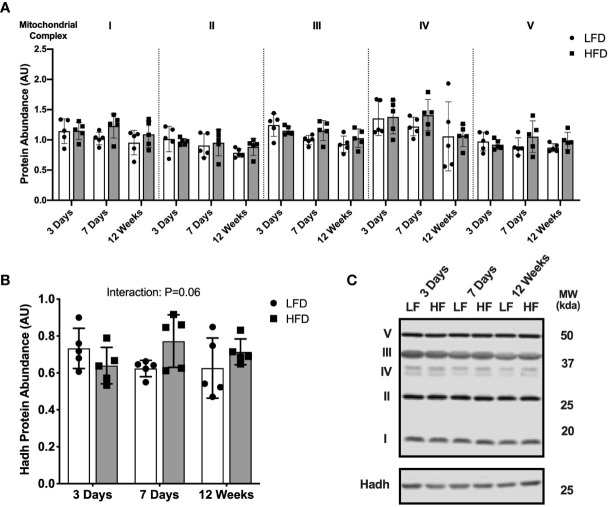
Short-term high-fat feeding did not alter mitochondrial enzyme abundance. Immunoblotting of lysates from quadricep muscle collected after a 4-hour fast from mice fed low-fat diet or high-fat diet for 3 days, 7 days, or 12 weeks. **(A)** Protein abundance of subunits in mitochondrial respiratory complexes I-V expressed in arbitrary units. **(B)** Hadh protein abundance in tissue lysates. **(C)** Representative blot images for **(A, B)**. Data are means ± SD; n=4-5. Two-way ANOVA was used to test for effects of diet and time. P values are main effects.

We next measured protein abundance of subunits found in the mitochondrial respiratory chain complexes to determine if the high-fat diet altered mitochondrial protein abundance. Mitochondrial complex protein abundance was not different between diet groups at any time point (**Figure 2A**). The abundance of 3-hydroxyacyl-CoA dehydrogenase (Hadh) was measured as an enzymatic marker of β-oxidation. There was a slight but non-statistically significant interaction effect between diet and time where Hadh abundance was slightly higher in the HFD group compared with LFD only after 7 days or 12 weeks (**Figure 2B**; interaction effect, p=0.06). Representative blot images are presented in **Figure 2C**.

Together, these data suggest short-term HFD causes a transient increase in ROS production which may activate an adaptive stress response. We next investigated mitophagy and membrane dynamics as potential stress response pathways to the acute high-fat challenge.

### Short-Term High-Fat Feeding Induces Accumulation of Mitophagy Receptors

**Figure 3:**
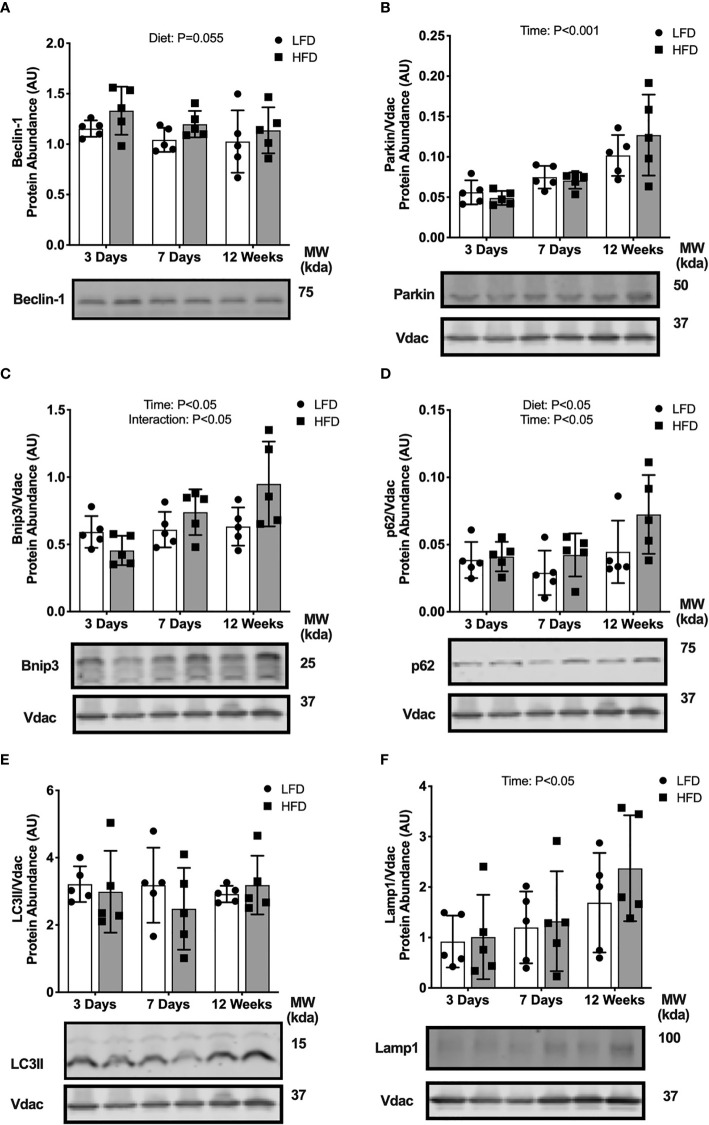
Short-term high-fat feeding induced accumulation of mitophagy receptors. Autophagy protein markers in skeletal muscle of mice fed low-fat diet or high-fat diet for 3 days, 7 days, or 12 weeks. Quadriceps muscles were prepped as whole tissue lysates for immunoblotting of Beclin-1 **(A)**. Gastrocnemius muscles were fractionated to prepare a mitochondrial-enriched fraction for immunoblotting of Parkin **(B)**, Bnip3 **(C)**, p62 **(D)**, LC3II **(E)** and Lamp1 **(F)**. Images below graphs are representative blot images. Protein targets in the mitochondrial fraction are normalized to Vdac abundance as a mitochondrial protein control. Panels **(C–E)** contain the same Vdac image because the targets are from the same membrane. Data are means ± SD; n=4-5. Two-way ANOVA was used to test for effects of diet and time. P values are main effects.

We considered if the high-fat challenge stimulated autophagosome formation or mitophagy in skeletal muscle. Beclin-1 abundance in the whole tissue was higher in the HFD mice compared with LFD (main effect of Diet; **Figure 3A**), suggesting greater activation of autophagosome formation. Protein abundance of parkin, a mitochondrial E3 ligase, was not different between diet groups at any time points (**Figure 3B**). The autophagy receptors, p62 and Bnip3, accumulate on the mitochondrial membrane to recruit autophagosomes through interaction with the autophagosome membrane protein LC3II. There was an interaction effect between diet and time where Bnip3 abundance in the mitochondrial fraction was lower after 3 days HFD compared with LFD, then higher after 7 days and 12 weeks of HFD compared with LFD (**Figure 3C**). Protein abundance in the mitochondrial fraction of p62 was greater in the HFD group compared with LFD and increased over time (main effects of Diet and Time; **Figure 3D**). Collectively, these data demonstrate activation of signals to promote autophagosome formation and recruitment to the mitochondria.

We considered if these upstream mitophagy signals would lead to changes in downstream autophagosome or lysosome markers. LC3II protein abundance in the mitochondrial enriched fraction was not different between diet groups at any time point (**Figure 3E**). The abundance of lysosome marker, Lamp1, also was not different between diet groups (**Figure 3F**). Together, these data indicate high-fat feeding increased upstream activation of mitochondrial autophagy receptors (Bnip3, p62) and autophagosome formation potential (Beclin-1), without changes to downstream autophagosome and lysosome abundance localized to the mitochondria.

### Short-Term High-Fat Feeding Alters Fusion But Not Fission Markers

**Figure 4:**
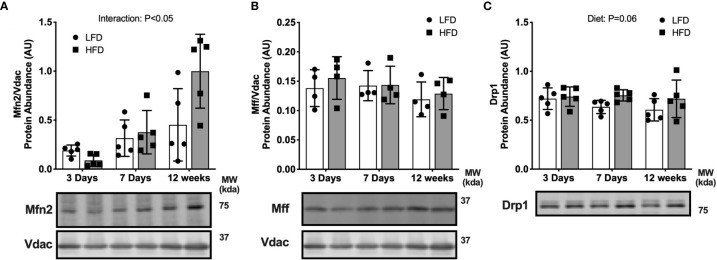
Short-term high-fat feeding altered fusion marker Mfn2. Fusion and fission markers in skeletal muscle of mice fed low-fat diet or high-fat diet for 3 days, 7 days, or 12 weeks. Quadriceps muscles were prepped as whole tissue lysates for immunoblotting. Gastrocnemius muscles were fractionated to prepare a mitochondrial-enriched fraction for immunoblotting of Mitofusion 2 (Mfn2) **(A)** and mitochondrial fission factor (Mff) **(B)**. Drp1 protein abundance in whole tissue lysate **(C)**. Images below graphs are representative blot images. Protein targets in the mitochondrial fraction are normalized to Vdac abundance as a mitochondrial protein control. Panels **(A, B)** contain the same Vdac image because the targets are from the same membrane. Data are means ± SD; n=4-5. Two-way ANOVA was used to test for effects of diet and time. P values are main effects.

We determined if high-fat diet altered regulators of mitochondrial fusion and fission, which regulate mitochondrial reticulum morphology in response to metabolic stress. There was an interaction effect between diet and time where Mitofusion 2 (Mfn2) was lower after 3 days of HFD compared with LFD but was greater after 12 weeks of HFD compared with LFD (**Figure 4A**). There were less pronounced differences in fission markers between HFD and LFD indicated by no difference in mitochondrial fission factor (Mff) abundance in the mitochondrial enriched fraction (**Figure 4B**). There was a slight but non-significant increase in dynamin related protein 1 (Drp1) in the HFD mice compared with LFD mice in whole tissue (main effect of Diet; **Figure 4C**). Together, these data suggest an initial decrease in fusion at 3 days, with no major changes to fission.

### Palmitate Treatment in C2C12 Myotubes Activates Mitophagy and Fusion/Fission

Our findings in mice suggest that short-term high-fat feeding increased autophagosome formation and mitophagy receptor abundance without a concurrent increase in autophagosome localization to the mitochondria. It is possible that the high dietary lipid triggered an autophagic stress response. We next used a cell culture model with an autophagy inhibitor (bafilomycin) to determine the effect of lipid stress on autophagy signaling and mitophagy flux.

**Figure 5:**
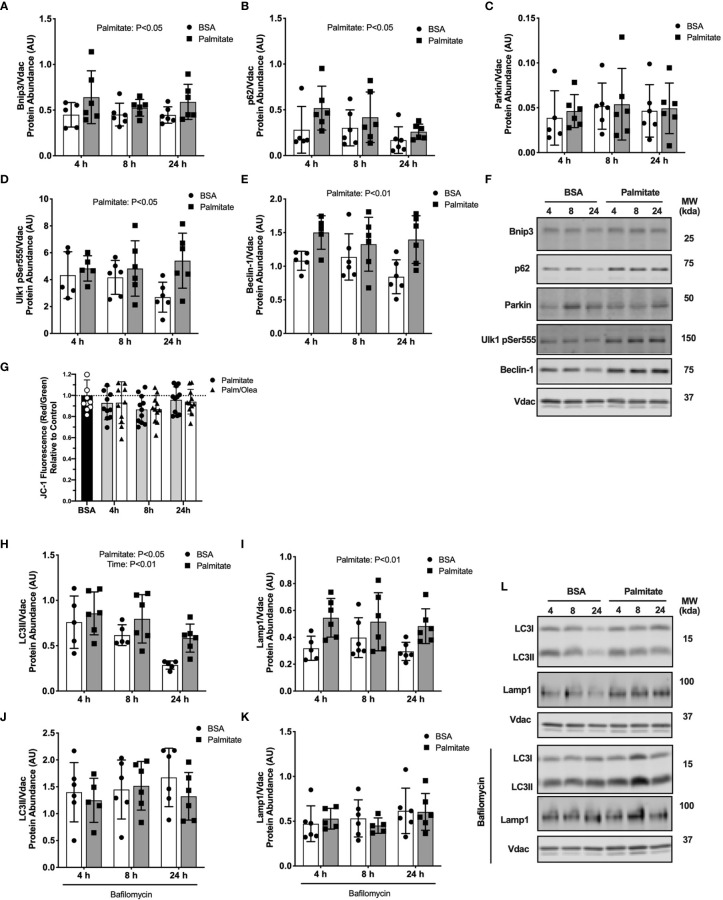
Palmitate treatment in myotubes activated mitophagy. Autophagy protein markers in C2C12 myotubes treated with palmitate for 4, 8, or 24 hours. Cell lysates were fractionated to prepare a mitochondrial-enriched fraction for immunoblotting of Bnip3 **(A)** p62 **(B)** Parkin **(C)** Ulk1 pSer555 **(D)** and Beclin-1 **(E)**. Representative blot images for **(A–E) (F)**. JC-1 fluorescence indicating mitochondrial membrane potential **(G)**. Mitochondrial localized LC3II **(H)** and Lamp-1 **(I).** Cells were treated with bafilomycin to inhibit autophagosome formation then mitochondria isolated and probed for LC3II **(J)** and Lamp1 **(K)**. Representative blot images for **(H–K) (L)**. Protein targets in the mitochondrial fraction are normalized to Vdac abundance as a mitochondrial protein control. Data are means ± SD; n=5-6 for immunoblots and n=11 for JC-1. Two-way ANOVA was used to test for effects of diet and time. Unpaired t-tests were used to test for JC-1 differences. P values are main effects.

Palmitate treated C2C12 myotubes had greater abundance of the mitophagy receptors Bnip3 and p62 in the mitochondrial-enriched pellet across all time points compared with BSA control (main effect of Palmitate; **Figures 5A, B**), suggesting a rapid and sustained increase due to palmitate. No difference in Parkin abundance was detected at any time points (**Figure 5C**). Similar to the mouse data, these data suggest mitophagy receptors accumulate in response to an acute lipid challenge without an increase in Parkin localization.

We next assessed upstream autophagy signaling in response to palmitate treatment. Phosphorylation of Ulk1 on Ser555 is an upstream activator of Beclin-1 to promote autophagy. Palmitate treated myotubes had greater Ulk1Ser555 phosphorylation and Beclin-1 abundance compared with BSA control at all time points (main effect of Palmitate; **Figures 5D, E**), suggesting sustained activation of autophagosome formation during lipid treatment. Representative blots images are presented in **Figure 5F**.

We considered if these upstream mitophagy signals would lead to changes in downstream autophagosome or lysosome markers. Palmitate treated cells had greater LC3II and Lamp1 abundance in the mitochondrial-enriched pellet compared with BSA control across all three time points (main effect of Palmitate; **Figures 5H, I**). However, treatment with autophagy inhibitor bafilomycin blunted the accumulation of LC3II and Lamp1 (**Figures 5J, K**), suggesting lower rates of autophagosome degradation during palmitate treatment. Representative blots images are presented in **Figure 5L**.

We considered declines in membrane potential (measured by JC-1 fluorescent probe) as a possible mitochondrial stress signal to activate mitophagy. Palmitate treatment for 8 hours decreased mitochondrial membrane potential compared with control cells (-14%, p<0.05; **Figure 5G**). There were no differences at 4 or 24 hours compared to basal, the latter indicating mitochondria recovered after the loss in membrane potential. The combination of palmitate and oleate had an identical effect to palmitate treatment alone on JC-1 signal (-13%, P<0.05; **Figure 5G**), suggesting total lipid rather than type of fatty acid drove the drop in mitochondrial membrane potential.

**Figure 6:**
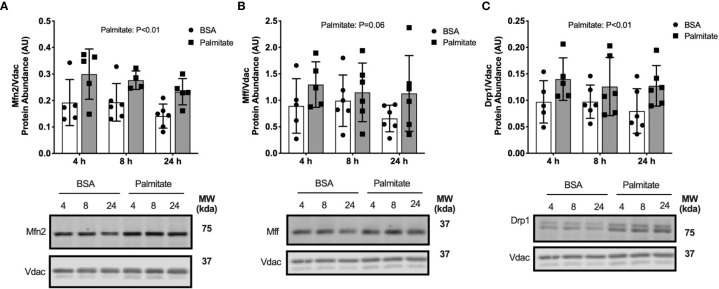
Palmitate treatment in myotubes activated fusion/fission. Fusion and fission markers in C2C12 myotubes treated with palmitate for 4, 8, or 24 hours. Cell lysates were fractionated to prepare a mitochondrial-enriched fraction for immunoblotting for Mitofusion 2 (Mfn2) **(A)**, mitochondrial fission factor (MFF) **(B)** and DRP1 **(C)**. Images below graphs are representative blot images. Protein targets in the mitochondrial fraction are normalized to Vdac abundance as a mitochondrial protein control. Panels **(B, C)** contain the same Vdac image because the targets are from the same membrane. Data are means ± SD; n=5-6. Two-way ANOVA was used to test for effects of diet and time. P values are main effects.

We determined if palmitate altered regulators of mitochondrial fusion and fission. Palmitate treated cells had greater Mfn2, Mff, and Drp1 abundance in the mitochondrial-enriched pellet at all time points compared with BSA control cells (main effect of Palmitate; **Figures 6A–C**), suggesting lipid treatment activated regulation of membrane dynamics.

Overall, the palmitate-induced activation of upstream mitophagy receptors and fusion/fission dynamics in C2C12 myotubes align with our data in mice demonstrating short-term high-fat feeding activated mitochondrial stress response pathways. Additionally, the use of the autophagy inhibitor in myotubes demonstrated that the high-fat condition suppressed autophagosome degradation, despite greater activation of upstream mitophagy signals.

### Hydrogen Peroxide Treatment in Myotubes Activates Mitophagy

**Figure 7:**
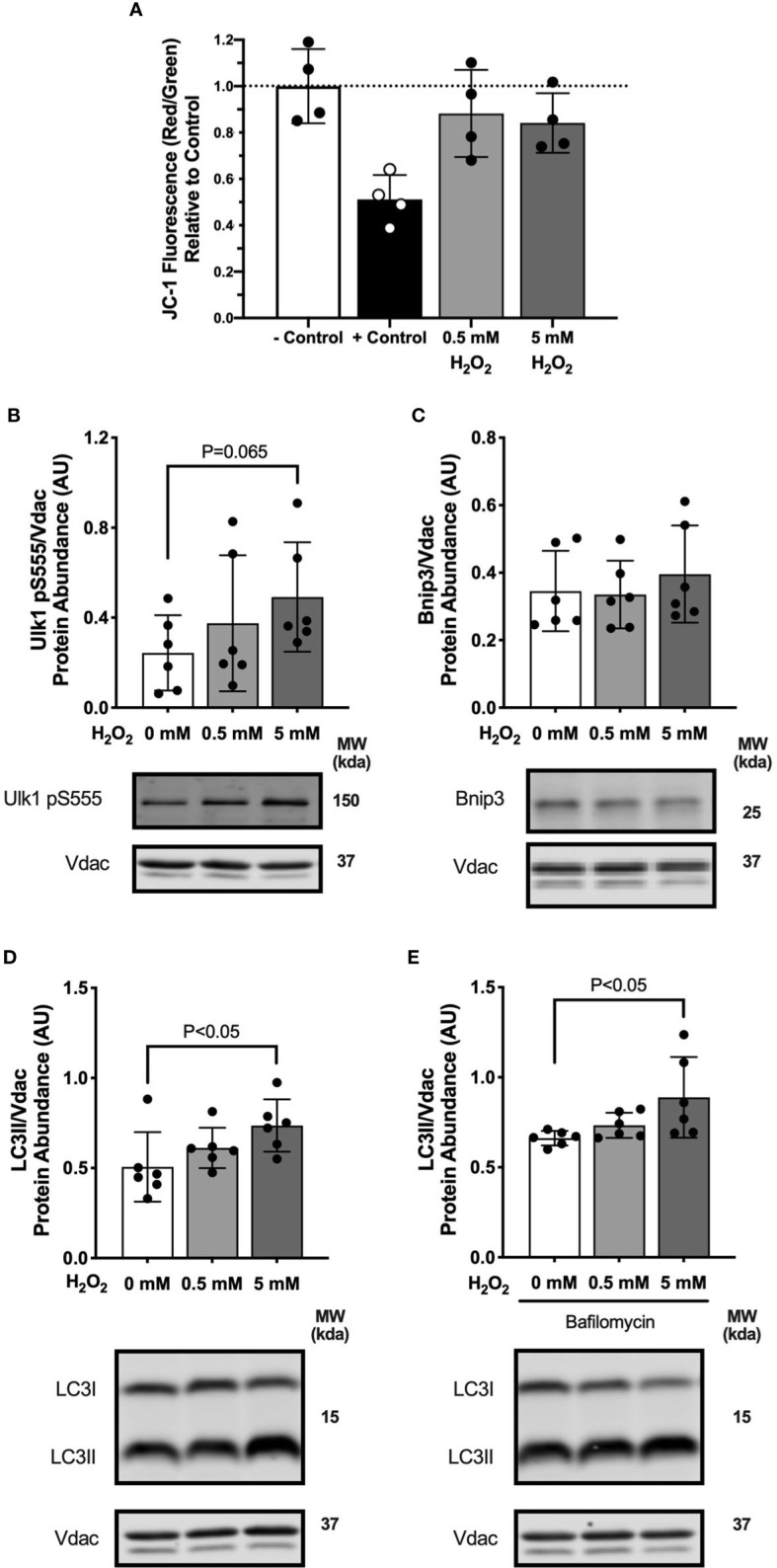
Hydrogen peroxide treatment in myotubes activated mitophagy. Autophagy protein markers in C2C12 myotubes treated with specified concentrations of hydrogen peroxide for 1 hour. Cell lysates were fractionated to prepare a mitochondrial-enriched fraction and cytosolic fraction for immunoblotting. **(A)** JC-1 fluorescence indicating mitochondrial membrane potential. – Control is DMED and + Control is FCCP. **(B)** Ulk1 pS555 protein abundance in mitochondrial fraction. **(C)** Bnip3 protein abundance in mitochondrial fraction. **(D)** LC3II protein abundance in mitochondrial fraction. **(E)** LC3II protein abundance in mitochondrial fraction in bafilomycin treated cells. Images below graphs are representative blot images. Protein targets in the mitochondrial fraction are normalized to Vdac abundance as a mitochondrial protein control. Panels **(D, E)** are from the same membrane. Data are means ± SD; n=6 for immunoblots and n=4 for JC-1. Unpaired t-tests were used to test for differences between groups.

We observed increased hydrogen peroxide production during lipid-supported respiration at 3 days in the high-fat diet fed mice. Therefore, we investigated reactive oxygen species (ROS) signaling as a possible mechanism for palmitate treatment to activate mitophagy in the myotubes. We used FCCP treatment as a positive control for decreasing membrane potential. FCCP is a strong protonophore that dissipates membrane potential and uncouples electron flow from ATP production. Two doses of H_2_O_2_ were added to the culture media to test the effects of ROS on membrane potential. A high dose of H_2_O_2_ induced a modest drop mitochondrial membrane potential compared with control (-16%, P=0.17; **Figure 7A**). Myotubes treated with 5 mM H_2_O_2_ had greater Ulk1pSer555 abundance compared with control (**Figure 7B**), suggesting an activation of mitophagy signaling. Bnip3 protein abundance was not different in H_2_O_2_ treated compared with control myotubes (**Figure 7C**). LC3II protein abundance was greater in myotubes treated with 5 mM H_2_O_2_ both without (**Figure 7D**) and with bafilomycin (**Figure 7E**) compared with control cells. Observations that H_2_O_2_ treatment activated mitophagy suggests palmitate-induced mitophagy may be partially mediated by increased ROS.

## Discussion

The purpose of this study was to investigate the initial effects of high dietary lipid on mitochondrial respiration and mitophagy in skeletal muscle. The main findings were that 3 days of high-fat feeding lowered the contribution of lipid-supported respiration relative to total capacity alongside greater ROS production, which resolved by 7 days. We hypothesized that the lipid overload to the mitochondria would activate mitophagy as an adaptive stress response. We detected higher autophagosome formation potential and greater abundance of mitophagy receptors after 7 days of high-fat feeding. These signals did not seem to transmit downstream as mitochondrial-localized autophagosome and lysosome abundance was not different between diet groups. Palmitate treatment in C2C12 myotubes demonstrated sustained signals to activate mitophagy but impaired autophagosome degradation during lipid treatment. These data suggest that several days of lipid overload activates upstream mitophagy signals, which may be mediated through ROS production, as an initial signal for skeletal muscle mitochondria to adapt to dietary lipid.

Our previous study demonstrated that compared with low-fat diet, mice consuming high-fat diet for 12 weeks had greater oxidative phosphorylation capacity specific for lipid substrates (6). In the current study, we demonstrated dynamic changes, specific to lipid respiration, within shorter time periods of 3 to 7 days. We assessed mitochondrial respiration with sequential additions of substrates and inhibitors to measure the combined response in oxygen consumption. The resulting flux rates indicated intrinsic oxidative phosphorylation and electron-carrying capacity of the mitochondrial respiratory system. Maximal oxidative phosphorylation or electron-carrying capacity were not different between diet groups at either of the short-term timepoints. However, the contribution of lipid-supported respiration to oxidative phosphorylation was lower after 3 days of high-fat feeding compared with the low-fat diet group. These findings suggest the initial response to lipid overload is a decrease in β-oxidation flux with a rapid recovery to control levels by 7 days. Other studies in isolated mitochondria demonstrate rapid intrinsic mitochondrial changes in response to high-fat feeding, such as increased lipid oxidation capacity by 1 week (26) or 3 weeks (27) in rats. Studies in permeabilized fibers of mice also revealed higher respiratory rates following high-fat feeding for 2 weeks (28) or 5 weeks (29). Our findings add to the knowledge of the time-course of skeletal muscle mitochondrial adaptations and indicate dynamic changes in mitochondrial lipid respiration within several days of high-fat feeding.

Fatty acid oxidation generates ROS in skeletal muscle mitochondria (17) which play a role in biological signaling (18). Our results demonstrated greater electron leak to hydrogen peroxide production during lipid-supported respiration after 3 days of high-fat feeding. However, after 7 days of high-fat feeding, electron leak was not different between diet groups. In the myotubes, palmitate treatment depolarized the mitochondria. Together, the *in vivo* and *in vitro* data are consistent with a minor oxidative stress on the mitochondria in response to lipids. A study using permeabilized fibers from rats demonstrated greater mitochondrial ROS production after 3 days of high-fat feeding and also at 3 weeks of high-fat feeding (16). The permeabilized fiber technique maintains a more intact redox environment that may reflect changes in antioxidant capacity that impacts ROS measurements. Our electron leak measurements were performed in isolated mitochondria and indicate electron coupling efficiency of the electron transport system. In agreement with our findings, 14 days of high-fat feeding increased mitochondrial coupling of mitochondria isolated from rat skeletal muscle (26). Lipid-induced ROS appears to stimulate greater mitochondrial coupling as a rapid and mitochondria-centric adaptation to high dietary fat.

The removal of mitochondrial proteins by mitophagy contributes to mitochondrial reticulum remodeling. Specifically, autophagy contributes to mitochondrial remodeling after skeletal muscle injury or exercise training to improve mitochondrial quality and respiratory function (11, 12). It is clear from KO models of autophagy that disruption of autophagy impairs mitochondrial structure and function (10, 30). Autophagy is also sensitive to nutritional factors where starvation activates autophagy to increase substrate availability for oxidative phosphorylation (31). We were interested in investigating the responsiveness of autophagy to a lipid stress as both a nutritional stress and a driver of mitochondrial adaptation in an intact autophagy system. Our results demonstrate high-fat feeding increases the accumulation of the mitophagy receptors Bnip3 and p62 beginning within 7 days. There was no difference between dietary groups in the abundance of autophagosome marker, LC3II, in the mitochondrial fraction. We measured abundance of mitophagy marker proteins which were the net result of activation and degradation and cannot distinguish flux in the mice. We performed cell culture experiments with an autophagy inhibitor, bafilomycin, to better capture the dynamic activation of mitophagy which allows for better interpretation of static protein abundance measurements. We observed greater abundance of Bnip3, p62 and Beclin-1, part of the protein kinase complex that activates autophagosome formation, in both mice and cells in the high-fat condition. Bafilomycin combined with palmitate treatment in cultured myotubes blunted accumulation of LC3II and Lamp1, suggesting lower rates of autophagosome degradation in the high-fat condition.

In agreement with our findings, palmitate treatment in cultured myotubes increased Bnip3 mRNA (32) and suppressed LC3II formation (33). Palmitate treatment also increased production of ROS which was associated with lower LC3II abundance in myotubes (34) and impaired autophagosome degradation in cardiomyocytes (35). Outside the context of lipid stress, mitochondrial uncoupling (36, 37) and increased mitochondrial ROS (20, 38) activated autophagosome formation and mitophagy mechanisms. Overall, high-fat conditions appear to increase mitochondrial stress and activate initial recruitment of mitophagy activators while inhibiting autophagosome clearance.

In addition to mitophagy, stress to mitochondria induces membrane remodeling through fusion and fission to alter size and shape of the reticulum. Observations in long-term high-fat diet rodent studies demonstrated suppressed fusion (Mfn2) (39, 40) and increased fission (Drp1) (40) resulting in more fragmented and smaller mitochondria with decreased respiratory function. Our mouse studies demonstrated fusion (Mfn2) decreased after 3 days of high-fat feeding then increased by 7 days, with no changes in fission (Mff, Drp1). Palmitate treatment in cells increased both fusion and fission markers. Contrary to our results, palmitate treatment in L6 myotubes increased Drp1 and decreased Mfn2 protein abundance resulting in greater fragmentation of the mitochondrial network (41). Our current mouse and cell culture studies support activation of both fusion and fission in response to short-term lipid stress, which may seem contradictory. Fission removes portions of the mitochondrial reticulum that have decreased membrane potential for degradation through mitophagy (42). Fission is also required for oxidative-stress induced mitophagy (19). However, mild depolarization of the mitochondrial membrane potential increased fusion of the reticulum into larger and more contracted structures to prevent further depolarization (43). Starvation also induces hyper-fusion of the mitochondria as a potential stress-response to promote ATP production (19). Therefore, acute lipid stress may activate both fusion and fission to promote removal of damaged mitochondria *via* autophagy while joining the mitochondrial network to prevent additional damage.

We acknowledge the limitations of using static measurements of autophagy protein markers rather than autophagic flux *in vivo*. We used multiple markers to distinguish various stages of autophagy/mitophagy to gain a more comprehensive understanding of pathway changes. Additionally, we measured proteins in both the whole tissue and a mitochondrial-enriched fraction of skeletal muscle to distinguish any differences in localization of the autophagy markers. We interpret our measures of autophagosome abundance as potential for autophagy rather than completion of autophagy. Additionally, the lack of morphological analyses of the mitochondrial reticulum is a limitation in our measurement of static fusion and fission markers.

An important consideration to discuss is the short-term time frame potentially captures an adjustment phase in the mice as they transition from chow diet to the experimental diet. The change in diet can cause a stress that could have resulted in less food consumption. We included a low-fat diet as a control group to help account for the stress induced by a change in diet in the high-fat diet group.

In conclusion, our study demonstrated that 3 days of high-fat feeding in mice impaired skeletal muscle mitochondrial fatty acid oxidation and increased ROS production that normalized by 7 days. The lipid-induced ROS production coincided with activated mitochondrial targeting for degradation through autophagy and mitochondrial fusion. Studies in C2C12 myotubes demonstrated impaired autophagosome degradation during lipid treatment. These findings increase understanding of short-term high-fat diet changes on mitochondrial respiratory function and support the role of mitophagy and membrane dynamics in mitochondrial adaptations to high-fat feeding.

## Data Availability Statement

The raw data supporting the conclusions of this article will be made available by the authors, without undue reservation.

## Ethics Statement

The animal study was reviewed and approved by Animal Care and Use Committee at Oregon State University.

## Author Contributions

SE performed majority of experiments, analysis, figure development and wrote the paper. HS, SN, and MR assisted with planning the study, performing animal procedures and data collection. All authors contributed to the article and approved the submitted version.

## Funding

This research was partially supported by the ACSM Foundation Doctoral Student Research Grant awarded to SE from the American College of Sports Medicine Foundation. MR was supported by K01DK103829 from the National Institutes of Health. SN was supported by KL2TR002370 as part of the Oregon Clinical & Translational Research Institute Clinical Translational Science Award UL1TR002371 from the National Institutes of Health.

## Conflict of Interest

The authors declare that the research was conducted in the absence of any commercial or financial relationships that could be construed as a potential conflict of interest.
